# From Allergy to Cancer—Clinical Usefulness of Eotaxins

**DOI:** 10.3390/cancers13010128

**Published:** 2021-01-03

**Authors:** Monika Zajkowska, Barbara Mroczko

**Affiliations:** 1 Department of Neurodegeneration Diagnostics, Medical University of Bialystok, 15-269 Bialystok, Poland; mroczko@umb.edu.pl; 2 Department of Biochemical Diagnostics, Medical University of Bialystok, 15-269 Bialystok, Poland

**Keywords:** CCL11, CCL24, CCL26

## Abstract

**Simple Summary:**

Eotaxins are small proteins included in the group of chemokines. They act mainly on blood cells called eosinophils which are involved in the pathogenesis of inflammatory processes. This connection leads to involvement of eotaxins in the pathogenesis of all inflammatory related diseases, such as allergic diseases and cancer. This paper summarizes the current knowledge about eotaxins, showing their usefulness as markers that can be used not only in the detection of these diseases, but also to determine the effectiveness of treatment.

**Abstract:**

Eotaxins are proteins which belong to the group of cytokines. These small molecules are secreted by cells that are mainly involved in immune-mediated reactions in the course of allergic diseases. Eotaxins were discovered in 1994 and their main role was considered to be the selective recruitment of eosinophils. As those blood cells are involved in the course of all inflammatory diseases, including cancer, we decided to perform an extensive search of the literature pertaining to our investigation via the MEDLINE/PubMed database. On the basis of available literature, we can assume that eotaxins can be used as markers for the detection and determination of origin or type of allergic disease. Many publications also confirm that eotaxins can be used in the determination of allergic disease treatment. Moreover, there are also studies indicating a connection between eotaxins and cancer. Some researchers revealed that CCL11 (C-C motif chemokine ligand 11, eotaxin-1) concentrations differed between the control and tested groups indicating their possible usefulness in cancer detection. Furthermore, some papers showed usefulness of eotaxins in determining the treatment efficacy as markers of decreasing inflammation. Therefore, in this paper we present the current knowledge on eotaxins in the course of allergic and cancerous diseases.

## 1. Eotaxins

Williams et al. [1] from the National Heart and Lung Institute in London in 1994 first described a new protein, which was able to selectively recruit eosinophils, without recruiting neutrophils, into sites of inflammation. This protein has been called eotaxin [2]. Subsequent research on this protein has confirmed its role as a potent eosinophil chemoattractant cytokine. Those researchers have also succeeded in describing its main receptor—CCR3 (CC chemokine receptor 3) [3,4,5]. Later, when following eotaxins were discovered, to facilitate their recognition they have been named with use of Arabic numbers (eotaxin-1, -2, -3) [6]. All eotaxins have the ability to bind to the previously mentioned CCR3 receptor. Interestingly, eotaxin-1 can also bind to some other receptors like CCR2 (CC chemokine receptor 2) and CCR4 (CC chemokine receptor 4), but its selectivity to CCR3 is highest of all of them [7]. Surprisingly, the gene for eotaxin-1 is clustered on the long arm of chromosome 17 (17q21.1–21.2) while the genes for eotaxin-2 and 3 are clustered on the long arm of chromosome 7 (7q11.23). Despite the differences in gene localization, they were classified into one group due to their similar functionality [5,8].

Eotaxins are proteins that can be produced by a wide range of cell types and are under dynamic control of multiple inflammatory mediators (Table 1) [9]. 

For example, eotaxin-1 (CCL11, C-C motif chemokine ligand 11) is produced by epithelial, endothelial, and smooth muscle cells; lung and dermal fibroblasts; alveolar macrophages; eosinophils; lymphocytes; adipocytes; and chondrocytes [8,9,10]. The main sources of eotaxin-2 (CCL24, C-C motif chemokine ligand 24) in the human body are fibroblasts, cutaneous and nasal epithelial cells, and macrophages [8,21]. Simultaneously, as eotaxin-3 has similar localization and functions to eotaxin-2, it is produced mainly by dermal fibroblasts and endothelial cells, as well as by HUVEC (human umbilical vein endothelial cells) treated with IL-4 (interleukin 4) [8,21].

CCR3 (C-C chemokine receptor type 3) is the main receptor for all eotaxins. The CCR3 gene is clustered on the short arm of chromosome 3 (3p21.3). This receptor can be found on the cell surface of white blood cells (especially eosinophils and basophils), mast cells, and activated T helper 2 lymphocytes. CCR3 can be also found on the airway epithelial cells. This receptor signaling induces actin polymerization; stimulates the migration, intracellular calcium dependent degranulation, and activation of eosinophils by G-protein-dependent mechanism; and release of toxic ROS (reactive oxygen species) [22,23]. 

Eotaxins are a potent stimulators of specific cell types. Eotaxin-1 is considered as a chemoattractant mainly for eosinophils, but also basophils, T helper 2 lymphocytes, thymocytes, macrophages, and mast cells. These activated by eotaxin-1 specific eosinophils are mainly implicated in allergic diseases (such as atopic dermatitis, allergic rhinitis, and asthma), parasitic infections, and inflammatory diseases characterized by eosinophil accumulation in tissues (eosinophilic esophagitis, gastroenteritis, and pneumonia). Additionally, CCL11 can be expressed in mucosa of gastrointestinal tract and may play a role in ulcerative colitis and other gastrointestinal disorders. Eotaxin-2, similar to eotaxin-1, is important in the recruitment of eosinophils, basophils, T helper 2 lymphocytes, and mast cells, while eotaxin-3 recruits killer lymphocytes and monocytes into sites of inflammation. Eotaxin-3 is also a chemoattractant for eosinophils and basophils and may contribute to the accumulation of eosinophils in atopic diseases [24,25,26,27,28,29,30,31,32,33,34]. Allergic diseases, in which all eotaxins are involved, belong to the group of inflammatory diseases. For example, in the case of asthma, the main mechanism of action is connected with tissue accumulation of eosinophils within the bronchial wall. Eosinophils contain secretory granules, which are connected with characteristic dark pink staining in standard hematoxylin and eosin preparations. Content of those granules can be released locally by several mechanisms. In tissues, activated human eosinophils undergo cytolysis, which includes extracellular expulsion of membrane-bound granules. Consequently, these granulocytes (which possess several properties that promote inflammatory processes), after degranulation, release eosinophil cationic protein and eosinophil peroxidase, which can damage nearby tissues. That is why eosinophils are thought to be important in the underlying bronchial hyperreactivity in asthma. Eotaxins can stimulate the migration of eosinophils from the bloodstream to tissues by acting on the CCR3, which is located on the white blood cells surface [8,35]. Therefore, it can be presumed that in all inflammatory diseases in which mainly eosinophils, but also basophils or T helper lymphocytes are activated, the concentration of these proteins increases. Malignant tumors are also one of these diseases. Although eosinophils have been described as associated with cancer over a century ago, their role in cancer is still undefined. Recent observations have reported that they reveal regulatory functions relative to other immune cells in the tumor microenvironment or direct cytotoxic functions against cancer cells, leading to anti- or pro- tumor activity [36]. Many researchers have shown that TATE (tumor-associated tissue eosinophilia) or degranulation of eosinophils is connected with improved prognosis in many types of tumors [37], such as colorectal cancer [38], esophageal and oral squamous cell carcinomas [39,40], bladder [41], or prostate cancer [42]. Interestingly, some studies about recruitment of eosinophils in tumors has shown that tissue infiltration by eosinophils can be mediated by factors that can be released from necrotic tumor cells [43,44,45] and some of them may be eotaxins.

## 2. Cell Recruitment

All eotaxins are associated with numerous allergic responses. Eotaxin-1 is responsible for recruiting not only eosinophils (EOS) and basophils (BASO), but also T helper 2 lymphocytes, thymocytes, macrophages, and mast cells (MC). Simultaneously, eotaxin-2 and -3 are responsible for recruiting only EOS and BASO [46]. As only BASO and EOS are associated with all three eotaxins, they will be taken into further, more detailed analysis. 

EOS are a type of white blood cells containing secretory granules also called secondary granules in the cytoplasm, which stain dark pink in standard hematoxylin and eosin (H+E) preparations. This is due to high cationic protein content which reacts with the acidic eosin dye. EOS are also a source for over 35 cytokines, chemokines, and growth factors (i.e., IL-4, IL-13). The specific mechanism of stored cytokine release from the granules remains still unclear. These cells are immune system cells that play an essential role in parasitic and allergic reactions. EOS are maturating in the bone marrow from the multi-potential CD34+ stem cell under the influence of cytokines, such as IL-5, IL-3, and growth factors (GM-CSF). When the maturation process is completed, they move into the bloodstream and pass to the tissues under the action of IL-5 and high eotaxin concentrations [35].

BASO belongs to the group of leukocytes, their cytoplasm contains granules, which stains blue in basic H+E preparations. These cells are maturating also in the bone marrow and under the influence of appropriate cytokines (i.e., IL-3, IL-4) transform from stem cells into basophils. They have the ability to phagocytosis and are a source of histamine (which dilates blood vessels and increases their permeability), heparin (reduces blood clotting), and serotonin. These substances are released under the influence of immunoglobulin E, mainly in the course of allergic reactions and anaphylaxis from secondary granules within BASO [47].

Eotaxins are able to activate a series of intracellular signaling cascades and can also affect the migration of EOS and BASO towards increased eotaxins concentration. This can lead to EOS and BASO recruitment into inflammatory sites and prolonged survival of those cells in response to released mediators resulting with tissue eosinophilia. In addition, the severity of many eosinophil-associated disorders is believed to reflect the extent of eosinophil activation in the tissues. EOS and BASO play an important role in the pathogenesis of allergic diseases (i.e., asthma and allergic rhinitis). EOS are also the main source of cytotoxic proteins and several growth factors responsible for tissue destruction and remodeling. This is evidence that eosinophil-derived mediators are strongly associated with the pathogenesis of inflammatory processes. The main stimuli for secretion of eotaxins appear to be, for example, early pro-inflammatory cytokines (Table 1). On the other hand, this stimulation can be also suppressed by some other mediators as dexamethasone (Table 1) which is relevant to the clinical effectiveness of inhaled glucocorticoids at decreasing the eosinophil-rich inflammatory exudate (i.e., in the course of asthma). Therefore, the selective blockade of axis between eotaxins and CCR3 could impair EOS recruitment, indicating a target for the treatment of all types of eosinophil-related disorders [30,35,48,49].

Both EOS and BASO differentiate and maturate in the bone marrow and then they migrate into bloodstream constituting about 1–6% and less than 1% of circulating leukocytes, respectively. Under some circumstances, upon their activation, BASO and EOS may leave the bloodstream and enter extravascular tissues. This is mainly connected with activation of these cells during inflammatory processes, in which we can include both allergy and cancer. The direct influence of pro-inflammatory substances connected with activation of these cells and high concentrations of eotaxins have a direct impact on the migration of these cells into the tissues [50]. EOS and BASO can be activated in pathological conditions such as allergic diseases (i.e., asthma, allergic rhinitis, atopic dermatitis, and food allergy), but also allergic reactions, infections, autoimmune disorders, and even cancer. All of these diseases may develop in different tissues and organs but with similar pathogenic mechanisms [50].

EOS are the most important cell population which can also interact by IgE-independent activation with mast cells (also activated in the course of allergic diseases) in late and chronic allergic reactions. Mast cells can release a large number of pro-inflammatory, immunoregulatory, and tissue regulatory mediators such as histamine, proteoglycans (heparin and chondroitin sulfate), neutral proteases, and many cytokines [51,52]. Moreover, the result of allergic reactions depends on many interactions which appear in the inflamed tissues between immune and structural cells. An analogous situation occurs in the cancer microenvironment, where inflammatory immune cells play a crucial role. It has been hypothesized that, in inflamed tissues, MCs can degranulate and release mediators from their granules. This process is connected with the ‘late phase’ of inflammation. In this phase, MCs can interact with EOS (inflammatory cells infiltrating tissues). EOS can persist throughout the late phase and even when the inflammatory condition becomes chronic. As MCs are still functional during the chronic stages of inflammation, the ‘cross-talk’ between MCs and EOS could be an important regulator of the inflammatory response [50,52,53]. 

Mast cells and EOS are also known to infiltrate some types of tumors but, due to the wide range of mediators which they are releasing, it is difficult to define their specific pro- or anti-tumor activity. Consequently, MCs and BASO are able to modify different aspects of cancer progression, for example, tissue remodeling, angiogenesis, and invasiveness, but also cooperating with other innate and adaptive cells. However, MCs are mainly associated with increased levels in other CC chemokines—MCPs (monocyte chemoattractant proteins) [54,55].

## 3. Pathophysiology of Eotaxin-Related Diseases

In order to go to the specific role of eotaxins in the course of allergic and neoplastic diseases, it is also necessary to mention the role of these proteins in their pathophysiology, which both in allergic and neoplastic diseases is connected with chronic inflammatory response. This process is connected with dysregulation and maladaptive response that involves active inflammation, tissue destruction, and attempts at tissue repair. Most often it arises from genetic predispositions, physical or ischemic injury, infection, or environmental exposures. Initial inflammatory processes are mediated by tissue resident macrophages and mast cells, leading to the production of pro-inflammatory mediators (IL-4, IL-5, etc.). The main effect of these mediators is to elicit an inflammatory exudate locally. This is subsequently connected with the presence of plasma proteins and white blood cells that are normally restricted to the bloodstream. These cells can gain access, through the postcapillary venules, to the extravascular tissues. The inflammatory cells (eosinophils, basophils, mast cells, Th 2 lymphocytes, etc.) are strongly correlated with release of eotaxins. This may lead to tissue remodeling, which is the next step of ongoing inflammation. This remodeling can involve long-term changes to the structural elements of the affected sites (such as increased vascularity) and substantial alterations in the barrier function of the affected epithelia. Additionally, high eotaxin expression in tissues is connected with tissue eosinophilia, as EOS migrate into the site of higher eotaxin concentration [56,57]. As the course of chronic inflammation is not fully understood, and in many publications both the serum or plasma concentration and tissue expression of eotaxins is high in inflammatory processes, it can be suspected that the activation of eosinophils and basophils secreting these chemokines occurs not only in tissues, but also in the bloodstream. Details on individual eotaxins in the course of allergic and neoplastic diseases are discussed in the following chapters.

### 3.1. Role of Eotaxins in Allergic Diseases

Most studies assessing the plasma/serum concentration or tissue expression of eotaxins concern changes after treatment in the course of various allergic diseases (i.e., asthma, atopic dermatitis, eosinophilic esophagitis, and chronic rhinosinusitis). There are only a few works concerning differences between study groups and their usefulness as markers of allergic diseases. For example, higher concentrations of eotaxin-2 and -3 were found in asthma patients when compared to healthy controls [58]. The same results were obtained for eotaxin-1, by Kalinauskaite-Zukauske et al. [59] in different types of asthma. The highest concentrations were found in serum of severe non-allergic eosinophilic asthma patients; lower but also high concentrations were found in serum of non-severe allergic asthma patients and the lowest concentrations were observed in a healthy control group. Differences between all mentioned groups were statistically significant. These results reveal that concentrations of all eotaxins, especially eotaxin-1, might be helpful not only in diagnosis of asthma, but also in determination of its type [59]. 

Moreover, it has been reported that CCL 26 (eotaxin-3, C-C motif chemokine ligand 26) tissue expression is significantly increased in patients with eosinophilic nasal polyposis. CCL 26 as well as other eotaxins are the key cytokines involved in eosinophil recruitment. Eotaxins also specifically activate the chemokine receptor CCR3 and contribute to the accumulation of eosinophils in atopic diseases. This may suggest that those proteins play a key role in the pathogenesis of such diseases and might be useful in differentiation between eosinophilic and non-eosinophilic diseases [60]. 

Lugogo et al. [61] reported that eotaxin-1 and surfactant protein A (SP-A), which is an important mediator in regulation of eosinophil activities and maintains airway homeostasis, are decreased in obese patients. This is due to elevation in those patients of TNF-α (tumor necrosis factor α) concentration, which is a proinflammatory protein contributing, according to the authors, to the blockage of SP-A secretion and the passage of eosinophils secreting eotaxin-1 from the tissues into the bloodstream. Based on research with the use of mouse tracheal epithelial cells with use of exogenous TNF-α, they revealed that increased local TNF-α may lead to impaired eosinophil resolution (connected with decrease in eotaxin-1 secretion in blood) and could contribute to the eosinophilic asthma phenotype due to the accumulation of these cells in lung tissues [61].

Interestingly, Noah et al. [62] revealed that diesel exhaust exposure is connected with activation of EOS and high eotaxin-1 concentration in nasal lavage fluid of allergic rhinitis patients, which may be especially affected by eosinophil recruitment and activation caused by pollution [62].

In addition, eotaxin-3 can be also useful as a marker for eosinophilic gastritis diagnosis. Shoda et al. [63] have developed and validated a diagnostic panel with use of biopsy and blood samples, where eotaxin-3 proved to be the best, both in tissue and blood, eosinophilic gastritis biomarker. These results showed that eotaxins can be useful in all eosinophil-related diseases [63].

Eotaxins, as chemokines involved in allergic reactions, have been used in many publications as biomarkers of treatment efficacy. Many researchers focused on studying these and other parameters before and after administration of the tested drugs to assess their effectiveness based on the obtained results of concentration or expression. 

For example, the ASN002 (oral JAK/SYK inhibitor) induces rapid and sustained improvements in cellular infiltrates, atopic dermatitis molecular pathways, and epidermal barrier abnormalities in patients with moderate-to-severe atopic dermatitis. In this study, eotaxin-3 after treatment with doses not lower than 40 mg of ASN002 showed a significantly lowered expression in lesional skin at days 15 and 29 compared with the baseline and placebo or lower doses groups. This could indicate the use of this new class of oral therapeutics in patients with atopic dermatitis as inflammation suppressor [64].

Additionally, eotaxin-2 and -3 concentrations were significantly higher in asthma patients treated with 200 mg of benralizumab (antibody against the alpha-chain of the interleukin-5 receptor) after 84 days of treatment [58].

Interestingly, dupilumab reduces concentration of eotaxin-3 in nasal secretions and eotaxin-1 concentration in polyp tissues of patients with chronic rhinosinusitis with nasal polyposis. These findings suggest that blockade of IL-4Rα (interleukin 4 receptor α) by dipilumab can inhibit IL-4/IL-13 (interleukin-4/interleukin-13) signaling. These results combined with suppression of underlying inflammation (downregulation of Th-2 response) in nasal polyposis leads to clinical benefits for patients [65].

On the other hand, Bond et al. [66] revealed that there were no significant differences in relative expression of eotaxin-2 gene in bronchoalveolar lavage fluid of horses with mild asthma treated with dexamethasone. This discrepancy might be related to a different drug mechanism of action (downregulation of TNF-α and IL-5) despite the same effect (inhibition of the Th-2 response) [66]. Additionally, non-significant results after treatment of acute bronchiolitis in infants with use of montelukast were obtained by Tahan et al. [67] where concentrations of eotaxin-1 did not differ between the groups that received and did not receive the tested drug [67].

Additionally, inhibition of miR-221-3p expression suppressed eotaxin-2 and -3 expression in mice BEAS-2B bronchial epithelial cells. Moreover, airway epithelial cells are involved in the pathogenesis of airway eosinophilic inflammation in asthma by expressing inflammatory cytokines (i.e., eotaxins) which play critical roles in recruiting eosinophils into the airway. These findings along with information that overexpression of miR-221-3p intensifies airway eosinophilic inflammation and enhances expression of both eotaxins may lead to conclusion that epithelial and sputum miR-221-3p may be a novel biomarker for airway eosinophilic inflammation in asthma [68].

Moreover, van Rhijn et al. [69] mentioned that fluticasone propionate significantly decreases the expression of genes encoding eotaxin-3 and eosinophil count in patients with eosinophilic esophagitis. Similar results were obtained by Rothenberg et al. [70] where tissue eotaxin-3 protein levels were decreased in all the subjects treated with QAX576 (inhibitor of human IL-13) and by Molina-Infante et al. [71] where proton pump inhibitor (PPI) treatment also downregulated esophageal eotaxin-3 expression. In addition, Krug et al. [72] demonstrated that no less than 200mg doses of BI 671800 (oral CRTH2 antagonist), twice a day, significantly inhibited eotaxin-1 concentrations in nasal fluid of allergic rhinitis patients. Likewise, Nair et al. [73] demonstrated that PUR003 (inhaled cationic airway lining modulator (iCALM)) significantly reduced the eotaxin-1 concentration in comparison to placebo group after allergen inhalation in asthma patients. These results may indicate that fluticasone propionate, QAX576, PPI, BI 671800, and PUR003, decrease inflammatory state by lowering inflammatory proteins’ expression. It also demonstrates the usefulness of eotaxins as markers of treatment effectiveness [69,70,71,72,73].

What is more, Kim et al. [74] revealed that 8-week therapy with Actinidia arguta extract significantly reduces the concentration of eotaxin-1 in asymptomatic subjects with atopy. These findings suggest that Actinidia arguta extract provides an alternative mechanism of regulating allergic response in which eotaxin-1 might be a marker of therapy success [74].

In addition, Scadding et al. [67] reported that in allergic rhinitis patients who undergo immunotherapy, eotaxin-1 nasal fluid concentrations, 8 h after nasal allergen challenge, were significantly lower than in patients who did not undergo the immunotherapy. Interestingly, the lower concentrations of eotaxin-1 observed in people who previously had contact with the allergen during therapy indicate that this parameter may be useful in the diagnosis or screening of allergic diseases due to its fast response to the first contact with the allergen [75].

Additionally, Wang et al. [76] reported that patients affected by eosinophilic chronic rhinosinusitis with nasal polyps treated with budesonide transnasal nebulization had significantly decreased eotaxin-1 expression which suggests that it might be also useful in determining the success of therapy [76]. Other researchers conducted similar research and confirmed that budesonide inhalation reduces eotaxin-1 release from circulating lymphocytes in asthma [77].

In addition, Stylianou et al. [78] pointed out that serum concentrations of eotaxin-1 were mostly higher in patients treated with allergen-specific immunotherapy when compared to non-treated patients and healthy subjects. It might be related to increased inflammatory response in the human organism and may differ significantly from eotaxin-1 expression in local polyp tissue. What is more, concentrations of eotaxin-1 decreased slightly during therapy, but most of this data was statistically non-significant [78].

It is also worth mentioning that work of Dupin et al. [79] proved that eotaxin-1 does not affect the migration of blood fibroblasts in chronic obstructive pulmonary disease patients and is not a part of potential mechanisms implicated in such recruitment [79].

### 3.2. Role of Eotaxins in Cancer

There are only a few original studies concerning the use of eotaxin concentration in cancer, especially as predictive markers. All of the found papers concerned only eotaxin-1, which proves that the potential of these parameters is not fully known and understood. Expanding the knowledge on eotaxins in the course of developing cancer is necessary, as evidenced by the results of studies published so far.

Among the works found, only one publication assessed the concentration of CCL11 in patients with colorectal [80] cancer. This study showed that the concentration of CCL11 in the control group was significantly lower than in the study group. Certainly, this may indicate the increased production of eotaxin-1 in the course of colorectal cancer. In addition, the determination of the concentration of other eotaxins and their receptor would be a perfect complement to the research carried out so far. Based on the obtained results, further studies on the concentration of those parameters in the course of other neoplasms are advisable to show whether the increase in concentration is related to specific types of neoplasms and their location, or to the development of cancer cells and the resulting inflammation.

Some authors also tried to check the usefulness of eotaxins as predictive markers in the course of treatment of various cancers. For example, Melisi et al. [81] proved that galunisertib (TGFβ receptor inhibitor), plus gemcitabine, significantly lowered the concentration of eotaxin-2 in pancreatic cancer patients. Unfortunately, the exact mechanisms to explain this condition are still unknown. However, these results indicate that inflammation- and remodeling-associated proteins (i.e., eotaxins) might be involved in such response [81].

Furthermore, Siva et al. [82] pointed out that serum concentrations of eotaxin-1 in non–small-cell lung cancer patients were lower after chemoradiotherapy when compared to radiotherapy itself and increased with time after the therapy in both cases. It proves the high ability to destroy cells involved in the production of eotaxins in the applied therapies.

Moreover, Argiris et al. [83] demonstrated that eotaxin-1 concentrations significantly changed after therapy with use of cetuximab (EGFR inhibitor) in head and neck cancer patients. What is more, Tsao et al. [84] revealed that low concentrations of eotaxin-1 in serum of non–small-cell lung cancer patients are associated with shorter progression-free survival after vandetanib treatment. 

Although cervical intraepithelial neoplasia is not a cancerous disease, Koshiol et al. [85] reported that eotaxin-1 levels were substantially higher in human papilloma virus (HPV)(+) than HPV(−) women with <CIN1 (cervical intraepithelial neoplasia). Likewise, Marks et al. [86] also found that carcinogenic HPV infections are connected with high eotaxin-1 concentrations.

Summarizing the current knowledge on eotaxins in cancer, we might suggest that currently CCL11 is the best candidate for a biomarker which may be used in the future not only in the detection of cancerous diseases (as a marker of ongoing inflammation) but also as a marker of treatment efficacy (as a marker od decreasing inflammation). Structured data concerning all eotaxins in different cancer types were summarized in Table 2.

## 4. Literature Search and Data Extraction

**Figure 1:**
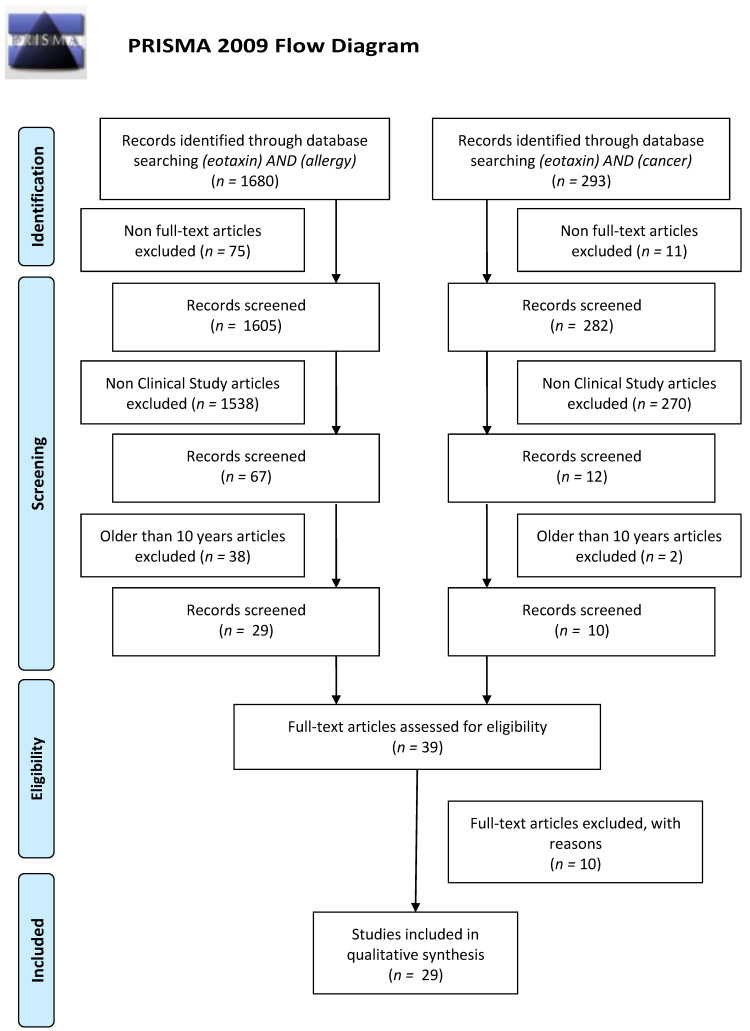
Schematic illustration of articles included in the review manuscript.

We performed a comprehensive literature search using the MEDLINE/PubMed electronic database on 29 July 2020 with the following search strategy: ‘(eotaxin) AND (allergy)’ and ‘(eotaxin) AND (cancer)’. In the first data search we found 1680 and 293 papers, respectively. Non-Full-Text articles were excluded. We have also applied two additional filters: Clinical Study as an Article Type and 10 years for Publication Date. In case of use of these data search exclusions we have found 29 and 10 papers, respectively. In the next step, we excluded all papers that were non-significant (papers not concerning allergic diseases, cancer, or eotaxins, i.e., papers concerning only healthy subjects (3 papers), non-allergic or non-cancerous diseases (1 publication), population differences (1 paper), or only CCR3 receptor (3 papers), and papers without necessary data, i.e., correlations without concentrations (1 paper) or papers in which the effect of exercise on cytokine levels in cancer survivors was studied (1 paper)). Finally, 22 and 7 (in sum 29) publications were included into the study, respectively. All steps were included in PRISMA 2009 Flow Diagram (Figure 1) [87].

## 5. Conclusions

Eotaxins are small proteins included into the group of chemokines that acts mainly on eosinophils and are involved in the pathogenesis of allergies. Reports from the last decade show that eotaxins can be used not only in the diagnosis of allergy as markers for the detection of allergic disease, but also to determine its origin (eosinophilic and non-eosinophilic) or type. Interestingly, some authors confirm that eotaxins can also be successfully used as indicators of the effectiveness of the allergic disease treatment. There are only a few studies indicating the usefulness of eotaxins in the diagnosis of neoplastic diseases or their use in the effectiveness of therapy, but their results are promising. On a basis of the results obtained so far, it may be suspected that eotaxin-1 (CCL11) could be a preferred eotaxin for cancer monitoring in the future. That is why further studies on the concentration of those parameters in the course of cancerous diseases and their therapy are advisable.

## Figures and Tables

**Table 1:** Inflammatory mediators which control the production of eotaxins [10,11,12,13,14,15,16,17,18,19,20].

Induction of Eotaxins Production	Suppression of Eotaxins Production
Th2-associated cytokines [11]	Th1-associated interferon (IFN-γ) [17,18]
interleukin 4 (IL-4) [19,20]	interleukin 17 (IL-17) [14]
interleukin 13 (IL-13) [20]	dexamethasone [10]
bacterial lipopolysaccharide (LPS) [20]	bisphosphonates [11]
tumor necrosis factor alpha (TNF-α) [19]	β2 adrenergic receptor agonists [12,13]
histamine [16]	fumaric acid [15]

**Table 2:** Usefulness of eotaxins in different types of cancer.

Cancer Type	Chemokine	Usefulness	Reference
Colorectal	CCL11	detection	[72]
Pancreatic	CCL24	treatment efficacy	[73]
Lung	CCL11	treatment efficacy	[74]
Head and neck	CCL11	treatment efficacy and shorter progression-free survival	[75,76]
CIN	CCL11	determination between HIV(+) and HIV(−) infections	[77,78]

## Data Availability

No new data were created or analyzed in this study. Data sharing is not applicable to this article.
